# Self-Measured Blood Pressure Monitoring: Program Planning, Implementation, and Lessons Learned From 5 Federally Qualified Health Centers in Hawai‘i

**DOI:** 10.5888/pcd17.190348

**Published:** 2020-06-25

**Authors:** David A. Stupplebeen, Catherine M. Pirkle, Tetine L. Sentell, Blythe M. I. Nett, Lindsey S. K. Ilagan, Bryan Juan, Jared Medeiros, L. Brooke Keliikoa

**Affiliations:** 1 Healthy Hawaiʻi Initiative Evaluation Team, Office of Public Health Studies, University of Hawai‘i at Mānoa, Honolulu, Hawaii; 2 Chronic Disease Prevention and Health Promotion Division, Hawaiʻi State Department of Health, Kapolei, Hawaii; 3 Hawaiʻi Primary Care Association, Honolulu, Hawaii; 4 Lānaʻi Community Health Center, Lānaʻi City, Hawaii

## Abstract

Self-measured blood pressure monitoring programs (BPMPs) are effective at controlling hypertension. We examined implementation of self-measured BPMPs at 5 Hawaiʻi-based Federally Qualified Health Centers (FQHCs). In a process evaluation of these programs, we found that FQHCs developed protocols for self-measured BPMP recruitment and enrollment and provided additional supports to account for their patients’ psychosocial needs to achieve blood pressure control, such as lifestyle change education and opportunities through referrals either to on-site or other programs (eg, on-site gym, tobacco cessation program). Common barriers across sites included insufficient material support for blood pressure monitors and data collection; funding, which affects program sustainability; and the lack of an “off-the-shelf” self-measured BPMP intervention. Policy makers and funding organizations should address these issues related to self-measured BPMPs to ensure implementation success.

**Summary**

**What is already known on this topic?**

Self-measured blood pressure monitoring programs (BPMPs) are effective in helping people with hypertension control their blood pressure.

**What is added by this report?**

This article explores the experiences of 5 Hawaiʻi-based Federally Qualified Health Centers (FQHCs) in implementing self-measured BPMPs. Because no nationally recognized self-measured BPMP curriculum existed at the time of this evaluation, the purpose of this article was to understand how FQHCs designed and implemented self-measured BPMPs in practice.

**What are the implications for public health practice?**

Policy makers, funding organizations, and intervention designers can draw on these experiences to make improvements to self-measured BPMPs in terms of support and toward the development of a standardized intervention curriculum.

## Background

Self-measured blood pressure monitoring programs (BPMPs) are interventions for patients to track their blood pressure at home or in other nonclinical settings. They are used to diagnose high blood pressure, improve blood pressure control, and reduce the risk of related conditions, including heart disease, heart attacks, and stroke (1). Compared with usual care, self-measured BPMPs can substantially decrease blood pressure versus usual care, especially when combined with additional support (2), including patient counseling (eg, medication management, lifestyle change), education on blood pressure management, or access to electronic monitoring tools (3). Program delivery can encompass team-based care and include telemonitoring with support from pharmacists or registered nurses (4,5). Implementing self-measured BPMPs in team-based care settings with other medical team members, such as community health workers (CHWs) (6), who work together with patients to achieve controlled blood pressure, is cost-effective (7).

## Purpose and Objectives

In 2014, the Centers for Disease Control and Prevention (CDC) awarded funds to the Hawaiʻi Department of Health (HDOH), Hawaiʻi Primary Care Association (HPCA), and 9 Federally Qualified Health Centers (FQHCs) to increase use of self-measured BPMPs with clinical support (8). In 2015, Hawaiʻi FQHCs served more than 150,000 patients, 42.8% of whom were Native Hawaiian or other Pacific Islander (NHOPI) (9). More than three-quarters of patients had incomes below the federal poverty level in 2013 (10). Although 17,883 Hawaiʻi FQHC patients had hypertension in 2015, only 64% had achieved blood pressure control (9). NHOPIs face socioeconomic barriers to hypertension management (11) similar to other populations who use FQHC services (12). At the start of the grant, there was no CDC-approved standardized curriculum for self-measured BPMPs; thus, FQHCs developed their own protocols and programs as part of their grant deliverables. In this article, we describe the self-measured BPMP components at 5 Hawaiʻi-based FQHCs during the grant period to highlight barriers and facilitators to program implementation.

## Evaluation Methods

Evaluators from the University of Hawaiʻi at Mānoa were contracted to provide a process evaluation that qualitatively assessed common self-measured BPMP components and that assessed barriers and facilitators at sites implementing the program. HPCA identified 5 FQHCs with self-measured BPMPs at varying levels of maturity; these FQHCs represented different practice settings (rural or urban) and patient population sizes (small or large). Health centers selected staff familiar with their self-measured BPMPs to participate in semi-structured video or telephone interviews, conducted in June and July 2018. Nine providers participated (Table 1), and all interviewees provided written consent. Evaluators asked how self-measured BPMP participants were identified, recruited, and enrolled; how programs were implemented; how patients were monitored; and about program barriers and facilitators. Four calls were recorded and transcribed; contemporaneous notes were taken during the fifth call. Transcripts and notes were qualitatively coded in Nvivo 11 (QSR International) and the primary evaluator (D.S.) deductively grouped codes into themes to mirror a typical programmatic logic model (ie, inputs, activities, outputs, and short-/long-term outcomes; see the CDC State Heart Disease and Stroke Prevention Program Evaluation Guide at www.cdc.gov/dhdsp/docs/logic_model.pdf). This evaluation was approved by the University of Hawai‘i at Mānoa institutional review board.

**Table 1:** Workflow of Self-Measured Blood Pressure Monitoring Programs at 5 Hawaiʻi Community Health Centers

Health Center Number/Location/Size^a^	Interviewees	% of Patients With Hypertension (2018)^a^	Activities		
			Recruitment	Intake, Program Delivery, and Follow-Up	Hypertension Education and Lifestyle Change
1. Rural/large	2 CHWs	27.8	• Recruitment: DPP, EHR • Referral: physician • Outreach: community, FQHC physicians	• Intake: readiness assessment and introduction • Enrollment location: office or home • Measurement training: office or in home • Monitor set-up: in home • Program length: target, 3–6 months • Log collection: office or home • Calculation: manual, entered into EHR for physician	• Counseling and goal setting • Physical activity: planning, off-site group activities (eg, hula, bicycle rides) • Diet: healthy eating • Referrals: DPP, care management
2. Urban/large	Program coordinator	16.0	• Recruitment: DPP • Referral: physician	• Intake: readiness assessment, introduction, hypertension education before enrollment (3 sessions) • Enrollment location: office • Measurement training: office • Monitor set-up: office • Program length: target, 5 months • Log collection: office • Calculation: manual	• Physical activity: planning, on-site trainer/gym • Diet: DASH^b^ education • Referrals: dietician, tobacco cessation
3. Rural/large	Pharmacist, 2 CHWs	25.7	• Recruitment: EHR • Referral: physician	• Intake: readiness assessment and introduction • Enrollment location: pharmacy • Measurement training: office • Monitor set-up: office • Program length: 3 months • Log collection: office once per month • Calculation: manual, entered into EHR for physician	• Physical activity: planning, on-site gym • Diet: nutritionist/dietitian referral • Incentive program: diet/physical activity–related incentives • Referrals: tobacco use cessation, sleep studies
4. Rural/small	APRN, physician	20.9	• Recruitment: EHR • Referral: physician	• Intake: readiness assessment and introduction • Enrollment location: office or home • Measurement training: office or home • Monitor set-up: office or home • Program length: unlimited • Log collection: at home or in office, transferred by tablet to health information system • Calculation: electronic, health information system	• Counseling and goal setting • Physical activity: planning, on-site wellness program • Diet: planning, *PILI ‘Ohana* (existing culturally adapted diabetes curriculum for Native Hawaiians and other Pacific Islanders)
5. Urban/large	Program coordinator	38.7	• Recruitment: EHR • Referral: physician • Outreach: patients and FQHC physicians	• Intake: readiness assessment and introduction • Enrollment location: office • Measurement training: office or home • Monitor set-up: office or home • Program length: 3 months • Log collection: at home or in office • Calculation: manual, entered into EHR for physician	• Physical activity: on-site group activities (eg, hula) • Diet: food demonstrations • *Ola Hou* lessons (culturally adapted existing self-measured blood pressure monitoring program curriculum) • Referrals: medication payment assistance

Abbreviations: APRN, advanced practice registered nurse; CHW, community health worker; DASH, Dietary Approaches to Stop Hypertension; DPP, National Diabetes Prevention Program classes; EHR, electronic health record; FQHC, federally qualified health center.

a A small health center had <10,000 clients on average during 2016–2018; large centers had ≥10,000 on average for the same period. Source: US Department of Health and Human Services, Health Resources and Services Administration (13).

b Source: National Heart, Lung, and Blood Institute (14).

## Results

Across the 5 FQHCs, the main program goals were to confirm a hypertension diagnosis and control blood pressure among those with diagnosed hypertension. The primary ways programs sought to achieve blood pressure control were through blood pressure monitoring and lifestyle change programs. We present the themes that emerged from interviews.

### Programmatic inputs and components

#### Inputs

Self-measured BPMP programs started at various times. One site started in September 2016 and 3 sites started in October 2016. The remaining site had an existing self-measured BPMP that started before the grant in 2015, and it used grant funds to maximize its community care model with CHWs. In addition to hiring support staff at all 5 centers, grant funds were used for additional program supplies (eg, log books). Interviewees said staff, existing program curricula related to blood pressure management, and patient-centered practices were important program inputs. All 5 FQHCs engaged CHWs or health educators in self-measured BPMPs, together with pharmacists, nurses, care coordinators, patient navigators, medical assistants, social workers, and/or nutritionists. The American Heart Association donated monitors, which facilitated the creation of a monitor loan program for patients who could not afford to purchase them, and provided educational materials. Other existing patient educational materials used by FQHCs included resources from HDOH, a culturally tailored intervention called *Ola Hou* (hula for hypertension), and the National Diabetes Prevention Program (NDPP). Patient-centered practices, like working with patients to develop individual goals for controlling blood pressure, were important. One provider said, “Shared decision-making is, I think, progressively getting more incorporated into the management of the team as well as the providers.”

#### Program eligibility

All FQHCs enrolled current patients with hypertension, although 3 sites also used their self-measured BPMPs to formally diagnose hypertension. FQHCs mainly used a systolic/diastolic threshold of 140/90 mm Hg to determine eligibility, and 1 center also used 150/90 mm Hg for its patients who were older than 60.

#### Participant recruitment

 All FQHCs developed workflows for recruitment, which included internal bidirectional referral systems and electronic health record (EHR) algorithms to identify patients with undiagnosed hypertension. Participants were also recruited via existing programs at FQHCs, such as NDPP classes (Table 1). One site recruited participants through community wellness fairs and screening events; nonpatients were asked to become patients at the FQHC, at which time primary care providers (PCPs) formally referred these patients to the self-measured BPMP. PCPs at other FQHCs also made referrals directly to self-measured BPMP staff; however, some program staff mentioned having to remind PCPs through meetings or other means that self-measured BPMP was an available resource.

#### Program intake and delivery

FQHCs used many of the same intake and enrollment procedures. Potential participants complete readiness assessments and program introductions with their PCP or self-measured BPMP staff assigned through the EHR. The level of patient assessment differed by site. One site asked permission of potential patients to schedule a time to explain the process. Another site conducted 3 different patient assessments because many of their clients had other underlying psychosocial issues, such as houselessness or mental illness: “We've had times . . . where [the patients] come in, and then they don't really know what they're here for. Then they don't want to do it.” After assessment, patients who were willing and able to participate were formally enrolled in the program.

At 4 FQHCs, patients signed a rental agreement for a loaner blood pressure monitor. A fifth FQHC provided reduced-price, Bluetooth-enabled monitors for purchase, so data could be transferred from the monitor directly into the clinic’s health information system. This clinic’s advanced practice registered nurse said, “We talk with the patient about the cost of the monitor being $35 and that it’s theirs. They can use it as much as they want, even that they could have 2 people use it in their household.” Enrollment and setup sites included both the FQHC and patient homes. Clinics encouraged participants to take their blood pressure twice per day, although some patients only measured once per day. For sites with loaned monitors, self-measured BPMPs were conducted for 3 to 6 months; the FQHC that sold monitors had no end date for its program. Staff at all sites trained patients on the use and proper placement of the monitor cuff, proper posture during a blood pressure reading, and how to record the reading. Patients often logged their blood pressure readings by hand, and these data were then collected by staff either in the office or at participants’ homes. Self-measured BPMP staff manually calculated average blood pressure and then entered the data into the EHR. Bluetooth-enabled monitors used at 1 site allowed all blood pressure readings of patients to be digitally stored and electronically collected by the site’s staff. PCPs and self-measured BPMP staff used the data to confirm hypertension or titrate medication as appropriate.

#### Hypertension education and lifestyle change

All 5 FQHCs included additional blood pressure education or lifestyle change components as part of their self-measured BPMPs. All sites provided diet-related education, including menu planning, food preparation demonstrations, referrals to nutritionists, or dietary information. Goal setting and motivational interviewing were also used by FQHCs to address barriers to lifestyle change and blood pressure monitoring. One site used its behavioral health team to address issues that affect patients’ weight and hypertension:

“We will utilize [behavioral health specialists] to meet with patients to discuss goals of wanting to lose some weight and some motivational cognitive behavioral therapy . . . to help with some patients with multiple chronic diseases. These patients sometimes also have some behavioral health issues that we need to address as well.”

Sites also reported adding in physical activity supports, including hula classes, group bicycle rides, and using on-site gyms or wellness programs. Some sites took advantage of existing on-site programs including NDPP classes, *Ola Hou*, tobacco cessation, or referrals to dietitians.

### Barriers and facilitators to implementing self-measured BPMPs

Various barriers to implementing self-measured BPMPs and how sites overcame them were discussed (Table 2). Technologic limitations and availability of monitors were partially overcome by use of donated monitors from the local chapter of the American Heart Association. Patient-related barriers, especially houselessness or mental illness, potentially limited participation in programs; some clinics lost contact with these participants. One staff member said, “At the beginning, we were giving out the monitors at the first appointment. That caused us to lose a lot of monitors, because people wouldn't come back.” Sites initiated readiness assessments and rental agreements to help with these issues.

**Table 2:** Barriers and Facilitators to Implementing Self-Measured Blood Pressure Monitoring Programs (BPMPs) at 5 Hawaiʻi-based Federally Qualified Health Centers

Category	Action
**Barrier**	
**Availability and limitations of blood pressure monitors**	
Monitors costly for patients, clinics Older monitors not Bluetooth-enabled, led to hand calculating blood pressure averages, which was time consuming	Used donated monitors, create monitor loan program for patients
**Patient-related issues**	
Disabilities, family, finances, houselessness, immigration status, fear of hypertension or worsening of condition, and transportation	Staff implemented readiness assessments to identify patients willing and able to participate Some sites implemented pre-enrollment education before distribution of monitors Institute monitor loan agreements Assist patients in their homes
**Staffing challenges**	
Provider turnover and other patient needs led to a lack of referrals	Self-monitored BPMP staff had to train refresh physicians to remind them of the service
**Systemic challenges**	
No single “out-of-the-box” self-measured BPMP program available Lack of integrated data management between monitors and electronic health records Uniform data system reporting Disagreement about hypertension diagnostic cutoffs led to delayed referrals at one center Funding and reimbursement for program sustainability	Staff constructed programs from existing materials, recommendations One site used Bluetooth-enabled monitors to transfer data to electronic health record
**Facilitator**	
**Systemic **	
Grant funding	Allowed sites to hire staff for self-measured BPMPs, access technical assistance to build programs
Shared technical assistance	Sites helped each other and shared tips and ideas
Existing resources	American Heart Association resources, other educational programs materials, capacity-building assistance
**Patient-related**	
Program word-of-mouth	Patients let others know about the program and availability of blood pressure monitors
Hypertension education	Patients helped diffuse information about hypertension to families/friends
**Clinical practice–related**	
Patient-centered/team-based approaches	Clinics used a variety of staff, including clinicians, pharmacists, and CHWs, who employed patient-centered approaches (eg, lifestyle change, home visits)
Integrating self-measured BPMPs into clinic practice	Integration of self-measured BPMPs into clinical workflows, including into the electronic medical record for referral and entering blood pressure readings

Abbreviations: CHWs, community health workers; BPMPs, blood-pressure monitoring programs.

Program reach was stymied by a lack of provider referrals because of competing demands. One staff member said, “I hear it from other programs, too, that they don't get a lot of referrals in general. From what I hear, it’s that [PCPs] have so many other things to do in a visit or whatnot, that this may not be their top priority type of thing.” Staff at 2 different sites mentioned that turnover of PCPs and self-measured BPMPs staff affected capacity, with one saying, “Staff turnover in the recent past has led to backlog of referrals . . . the maximum capacity is 2 patients per day.” One site had started their program using an in-house pharmacist; however, the main funding for that position ended, and program operation was moved to other health education staff. Turnover, although challenging, was partially addressed through presentations of self-measured BPMPs to new PCPs.

In addition to other systemic barriers, interviewees frequently mentioned the lack of an “out-of-the-box” self-measured BPMP curriculum, which led program staff to combine materials from a variety of sources. Systemic facilitators included funding to initiate self-measured BPMPs, technical assistance and shared capacity-building with other implementing sites, and availability of existing educational materials. Another barrier mentioned was the lack of an agreed-upon hypertension diagnosis standard (2,15) among PCPs. One clinic received additional capacity-building assistance on self-measured BPMPs from clinicians who had previously developed a self-measured BPMP (4). Patient word-of-mouth about the program helped spread information about hypertension and encouraged others to participate. Lastly, the patient-centered and team-based care models used by FQHCs and integration of self-measured BPMPs into clinic workflows were important facilitators, which have been effective in other studies (5).

## Implications for Public Health Practice

This process evaluation identified several lessons learned and potential recommendations for policy makers and funding organizations. Foremost, recruitment, scaling, and sustainability were limited by the lack of material supports (eg, monitors) for program implementation, and staff turnover was a major barrier. Funding for other self-measured BPMP positions, like CHWs, is often grant-based, which can lead to burnout and contribute to turnover (16). Four FQHCs limited their program duration because they loaned monitors to patients who could not afford them, while the fifth site performed continuous monitoring, because patients purchased the monitors and because hypertension is a chronic condition. Manual calculation and entry of blood pressure readings into EHRs was a time-consuming process. Data management difficulties hindered further evaluation of the effectiveness of self-measured BPMPs and highlighted the importance of improving the ease and quality of data collection for both patients and providers. 

Funding organizations should address the lack of material resources, challenges to remote data collection and monitoring, program reimbursement, and the need for cost-effective health information technology to improve self-measured BPMP uptake and support program sustainability, especially for organizations with populations like those served by FQHCs. In 2018, Hawai‘i FQHCs served 157,097 patients, of whom 22,509 had hypertension; of those, 39% had yet to achieve blood pressure control (17), demonstrating the ongoing need for self-measured BPMPs. Second, patients’ needs or more urgent health matters interfered with participation in self-measured BPMPs; this was compounded by the lack of an “off-the-shelf” self-measured BPMP curriculum. To address this, sites first assessed patients individually for participation readiness to ensure patients were able to succeed. Second, sites compiled materials from existing programs on dietary and behavior modifications to educate participants on lifestyle changes to manage blood pressure. Then sites provided instrumental supports, such as opportunities for exercise or leveraging existing lifestyle-change programs. Lastly, FQHCs’ team-based care model involved multiple layers of staff to help manage self-measured BPMPs participants and their needs, such as CHWs going to participant homes for monitor setup and data gathering. We were unable to assess whether these social supports or patients’ own motivation were contributors to self-measured BPMP enrollment. We were also not able to assess whether differences in manual or Bluetooth-connected monitors, the primary instrumental support provided in these programs, affected compliance and program adherence by participants. Future research should examine these factors. Funders and policy makers should convene sites to provide input on their self-measured BPMP implementation experiences to help develop an off-the-shelf program based on lessons learned.

Five Hawaiʻi-based FQHCs implemented self-measured BPMPs that strategically addressed patients’ psychosocial and health needs. Systemic barriers hindered the sustainability of self-measured BPMPs at some sites and access to data, which hindered an outcome evaluation of these efforts. Policy makers should consider developing off-the-shelf self-measured BPMPs and provide material support to implementation sites through blood pressure monitor reimbursement and further financial support to maintain clinic staff.
